# Identification and characterization of new miRNAs cloned from normal mouse mammary gland

**DOI:** 10.1186/1471-2164-10-149

**Published:** 2009-04-07

**Authors:** Nezha Sdassi, Licia Silveri, Johann Laubier, Gaëlle Tilly, José Costa, Sarah Layani, Jean-Luc Vilotte, Fabienne Le Provost

**Affiliations:** 1 INRA, UR 339, Laboratoire de Génétique biochimique et Cytogénétique, F-78 350 Jouy-en-Josas, France

## Abstract

**Background:**

MicroRNAs (miRNAs) are small non-coding RNAs that have been found to play important roles in silencing target genes and that are involved in the regulation of various normal cellular processes. Until now their implication in the mammary gland biology was suggested by few studies mainly focusing on pathological situations allowing the characterization of miRNAs as markers of breast cancer tumour classes. If in the normal mammary gland, the expression of known miRNAs has been studied in human and mice but the full repertoire of miRNAs expressed in this tissue is not yet available.

**Results:**

To extend the repertoire of mouse mammary gland expressed miRNAs, we have constructed several libraries of small miRNAs allowing the cloning of 455 sequences. After bioinformatics' analysis, 3 known miRNA (present in miRbase) and 33 new miRNAs were identified. Expression of 24 out of the 33 has been confirmed by RT-PCR. Expression of none of them was found to be mammary specific, despite a tissue-restricted distribution of some of them. No correlation could be established between their expression pattern and evolutionary conservation. Six of them appear to be mouse specific. In several cases, multiple potential precursors of miRNA were present in the genome and we have developed a strategy to determine which of them was able to mature the miRNA.

**Conclusion:**

The cloning approach has allowed improving the repertoire of miRNAs in the mammary gland, an evolutionary recent organ. This tissue is a good candidate to find tissue-specific miRNAs and to detect miRNA specific to mammals. We provide evidence for 24 new miRNA. If none of them is mammary gland specific, a few of them are not ubiquitously expressed. For the first time 6 mouse specific miRNA have been identified.

## Background

Numerous small non-coding RNAs of 18–25 bases in length, called microRNAs (miRNAs), have been found to play important roles in silencing specific target genes. Recently Vasudevan *et al*. [1] have shown that miRNAs can also activate gene expression, inducing translation up-regulation of target messager RNAs (mRNAs) on cell cycle arrest. The total estimated number of reasonably conserved miRNAs in vertebrates varies from 250 [2] to 600 [3]. In human, Bentwich *et al*. [4] suggested that the total number of miRNAs is above 800. The sequences of many miRNAs are conserved among distantly related organisms [5], but recent evidences demonstrated the presence of primate-specific miRNAs [6,7]. miRNAs are transcripts which are cleaved from a ~70 nucleotides hairpin precursor by Dicer [8,9]. They regulate gene expression at the posttranscriptional level through binding to their target mRNAs by base-pairing and subsequently inducing either translational repression or mRNA destabilization [10]. miRNAs are involved in the regulation of various cellular processes, including cell differentiation, cell proliferation, development and apoptosis [11].

Several methods are used to characterize the miRNA expression profiles in specific tissues such as Northern blotting, RNase protection assay, RT-PCR and microarray analyses. All these approaches depend on the prior knowledge of the miRNA sequences. If the accurate profiling of known miRNA expression represents an important tool to investigate physiological and pathological states, the discovery of new miRNAs is still important. Bioinformatics' strategies and miRNA gene prediction algorithms have been used to screen genome sequences and to identify potential miRNAs [[2], for review [12]]. Schematically, the bioinformatic' approaches scan genomic sequences for the phylogenetic conservation of short nucleotides motifs located within genomic stretches that have the structural characteristics, ie secondary structures, of miRNA precursors. However such gene predictions may not reveal all miRNAs, and might especially miss those that are not phylogenetically conserved. Furthermore, all these *in silico *predictions require independent experimental validations. In contrast, the cloning approaches allowed the identification of miRNAs without prior knowledge of their sequences [for example [13]], but limit the identification only to those miRNAs present at specific moments in the studied organ.

The mammary gland is a dynamic organ whose structure changes throughout the female reproductive cycle. These successive physiological stages, that are regulated by hormones, growth factor ligands, their receptors and some transcriptional factors, are characterized by proliferation, differentiation and apoptosis of the mammary epithelial tissue which is embedded in the stroma [for review: [14,15]].

An implication of miRNAs in mammary gland biology was suggested by few studies mainly focusing on pathological situations, such as the appearance of breast cancer [[16] for review]. Some miRNAs were found deregulated in human breast cancers [17-21]. Recently the role of specific miRNAs (miR-206, -221 and -222) in the regulation of the human Estrogen Receptor-α in breast cancer cell lines has been demonstrated [22,23]. Moreover some miRNAs expression profiles have been used to identify human breast cancer tumor subclasses [24,25]. In the normal mammary gland the expression of known miRNAs has been studied in human [26] and mouse [27,28] at different developmental stages and found to be regulated. Moreover the identification and characterization of miRNAs from the bovine mammary tissue by Gu *et al*. [29] allowed the cloning of 33 distinct miRNAs, including 3 novel ones. Recently, Ibarra *et al*. [30] showed that several miRNAs are involved in the maintenance of mouse mammary epithelial progenitor cells. The full repertoire of miRNAs expressed in one tissue, in a specific condition or in specific cell types is not yet available and could revealed specific miRNAs. The mammary gland which is an evolutionary recent organ is a good candidate to search for such tissue-specific miRNAs.

## Results and discussion

### Cloning of new miRNAs from mouse mammary gland

Using the small RNA cloning method described by Lagos-Quintana *et al*. [31], cDNA libraries were constructed from RNA samples of mouse mammary glands collected at different stages (virgin 8 weeks, gestation 2-, 6- and 18-days, lactation 4-days and involution 1-day). A total of 455 clones were obtained and sequenced, of which 169, corresponding to 120 non redundant sequences, had inserts ranging between 18 and 28 nt in length (Additional file 1). Most of the remaining clones contained inserts smaller than 17 nt and were not further analysed. The 120 non redundant sequences were compared with known miRNAs by searching the miRBase database (release 12.0). Nine sequences matched with mature miRNAs, 3 of them perfectly with mouse let-7b (MG030), let-7c (MG070) and human miR-923 (MG041) respectively, 3 with a 1-nt difference with mouse miR-126-5p (MG034), miR-429 (MG018) and human miR-1268 (MG068), and 3 have partial homologies (Additional file 1). Human miR-923 is 100% homologous with the mouse genomic sequence. The above-mentioned 3 sequences that perfectly matched with known miRNAs were thus considered as corresponding to true miRNAs and not further analysed. Thirty-three sequences have partial homologies with 1 or several precursors of different species, among them 17 have partial homology with the miRNA strand, 8 with the miRNA* strand and 8 with the loop (Additional file 1).

One important criteria that distinguishes miRNA from other endogenous small RNA is the ability of their genomic flanking sequences to adopt a hairpin structure with the mature miRNA properly positioned within one of its strand in order to be excised during Dicer processing [32]. The 117 sequences cloned were aligned with the mouse genome using BLAST, 31 of them were not found to be homologous. They were discarded; and considered as being cloning artefacts. For some of them we cannot exclude that the mismatches observed are due to RNA editing [33]. Among the 86 homologous sequences, 15 showed homology with a single location, whereas the others could be located in several genomic regions, leading overall to a total of 441 localisations. To assess which of the 441 regions correspond to potential miRNA genes, their secondary structures were studied using the RNA folding program MFOLD [34]. One hundred and fourteen chromosomal regions, which correspond to 40 cloned sequences, could form a stable hairpin structure. Among the 114 chromosomal regions, 57 were annotated as ribosomal RNA or small nuclear RNA and were discarded. The 57 remaining corresponded to 33 cloned sequences.

**Figure 1:**
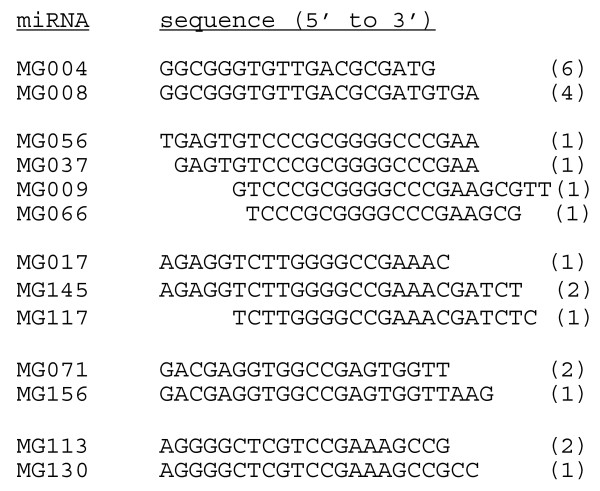
**Sequence variations of cloned mouse miRNAs**. Two to 4 variants were identified for 5 distinct families. The number of clones for each variant is indicated in the parentheses beside the sequence. The sequences of the corresponding precursors are presented in Additional file 2. For several families, more than one predicted precursors were determined by bioinformatics' analysis (Additional file 2).

**Figure 2:**
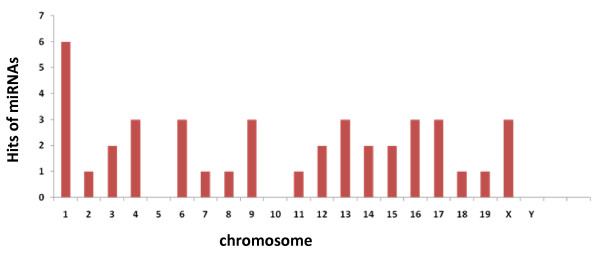
**Chromosomal distribution of the miRNA genes identified in this study**. The number of hits represents the number of miRNA genes localized on each chromosome. Thus study did not reveal the occurrence of a specific chromosome encompassing most of the mammary-expressed miRNA loci.

Their chromosomal locations and sequence alignments revealed that several cloned sequences were partially overlapping and could derive from the same precursor. In our study 5 families, corresponding to 13 sequences, were detected (Figure 1). Recently, the presence of variants has been reported after the characterization of cDNA libraries of small RNAs from porcine fibroblast cells [35] and from bovine adipose tissue and mammary gland [29]. The 3'-end variants may thus arise from preferential degradation at the 3' end or from imprecise processing of miRNA precursors by Dicer, thereby generating miRNAs with differing 3' ends [36,37]. However, we cannot exclude the possibility that these miRNA variants originate from multiple genomic loci. The functional specialization of miRNA variants is still unknown. The 33 cloned sequences could derive from 41 precursors (Additional file 2). In fact one cloned sequence could derive from several precursors (Additional file 2, for example MG141 could be issue from 3 precursors). In some cases one cloned sequence possess i) 2 identical precursors localised in 2 different chromosomes (Additional file 2, MG016-13 and -16), ii) 3 identical precursors clustered in one chromosome (MG055) or iii) 2 similar precursors (with 1 or 3 different nt) in two different chromosomes (Additional file 2, MG009/MG037/MG056/MG066-06 and -17, MG016-04 and -1 or MG141-12 and -15, for example). Overall the precursors are distributed on all the chromosomes but chromosomes 5, 10 and Y (Figure 2). Chromosome 1 appears to contain more mammary gland miRNA genes than any other chromosome. Our result are in agreement with published results [38,39] that showed that miRNA genes are distributed among all the chromosomes but chromosome Y. Twenty-six of the precursors are localised in intergenic regions, 14 in genes (intron: 11, 5'UTR: 1, 3'UTR: 1 and exon: 1) and 1 corresponds to one miRNA non-fully characterised (ENSMUSG00000076325) (Additional file 2). Thus the majority of miRNA genes are part of intergenic sequences (62%) as observed by Ro *et al*. [40]. By analysing the proximity of the 57 chromosomal locations in the mouse genome, 4 miRNA clusters were observed (Table 1). The miRNA genes are in the same cluster if they are less than 1000 bp apart on the same chromosome [29]. This physical proximity is consistent with recent reports of miRNA clustering within the human genome [41]. Among the 4 clusters, 2 correspond to the association of two new precursors, MG016-01 and MG016-16, with 2 miRNAs already described mmu-miR-689-1 and mmu-miR-689-2, respectively (Table 1). The cluster MG055 on chromosome 1 has three repeats of the same precursor.

**Table 1:** Four new miRNA gene clusters.

cluster	chromosome locations
MG016 and mmu-miR-689-1	Chr 1: 169270471–169270525 and 169270532–169270640

MG055a; b and c	Chr 1: 172996751–172996851, 173004422–173004522, 173011996–173012096

MG113/MG130; MG141	Chr 12: 1008832575–100883333, 100883323–100883403

mmu-miR-689–2 and MG016	Chr16: 11144133–11144241 and 11144248–11144304

The evolutionary conservation of the 33 new miRNAs (25 distinct miRNAs + 8 variants; Table 2) was studied by comparison of their sequences with the human, rat, dog, monkey, chicken, Danio rerio, monodelphis and Ornithorhynchus anatinus genome sequences. Only 3 were conserved in all the species studied. Six were not detected in any other species (Table 2). If the phylogenetic conservation of the miRNA sequence is one of the criteria established by Ambros *et al*. [32] to characterize miRNA, it is important to recall that for some species the sequencing of their genome is not complete enough to detect all the sequences cloned here. It remains that the bioinformatics' analysis performed in this study has allowed identifying mouse specific miRNA.

**Table 2:** Conservation of the 33 new miRNAs in other species.

**name**	**Ra**	**Hu**	**Do**	**Or**	**Mo**	**Ch**	**DR**	**Md**
MG004/MG008	0	x	0	0	x	x	x	0
MG009/MG037/MG056/MG066	0	0	x	x	0	x	0	x
MG013	0	x	x	x	x	x	x	x
MG016	0	0	0	0	x	0	0	0
MG017/MG117/MG145	0	x	0	0	x	x	0	0
MG023	0	0	0	0	0	0	0	0
MG039	0	0	0	0	0	0	0	0
MG053	0	0	0	0	0	0	0	0
MG054	x	x	0	0	x	x	0	x
MG055	x	x	x	0	x	0	x	x
MG057	0	0	0	0	0	0	0	0
MG059	x	x	0	0	x	0	0	x
MG071/MG156	x	x	x	x	x	x	x	x
MG112	x	x	x	0	x	x	x	x
MG113/MG130	0	0	0	0	0	0	0	0
MG119	x	x	x	0	x	x	0	0
MG121	x	x	x	x	x	x	x	x
MG123	x	x	x	x	x	x	x	x
MG125	x	x	x	x	x	x	0	x
MG127	x	x	0	0	x	x	0	x
MG141	0	0	x	0	x	0	0	0
MG143	0	0	0	0	0	0	0	x
MG144	x	x	0	0	x	x	x	x
MG147	0	x	x	0	x	0	x	x
MG155	x	x	x	0	x	x	x	x

0: non detected, x: detected

Ra, rat; Hu, human; Do, Dog; Or, ornithorhynch; Mo, monkey; Ch, chicken; DR, Danio Rerio; Md, Monodelphis.

### Expression profiling of the 33 new miRNAs

**Figure 3:**
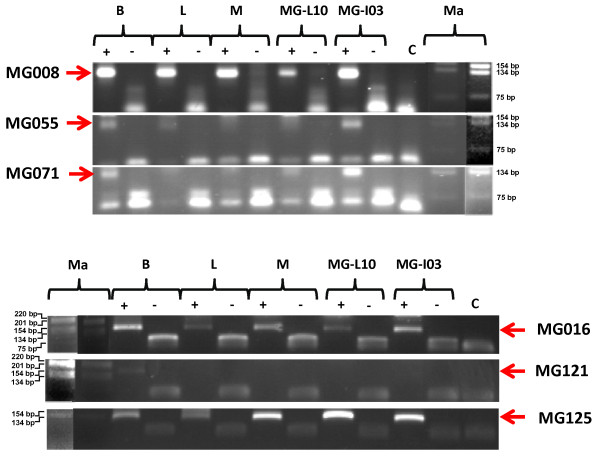
**Expression of new miRNAs in different tissues**. RT-PCR analysis (+, with reverse transcriptase; -, without reverse transcriptase) of newly miRNAs in brain (B), liver (L), muscle (M) and mammary gland (MG) during lactation (day-10, L10) and involution (day-3, I03) were presented for 6 miRNAs. C samples without cDNA. ma: DNA marker. It is the 1-kb ladder from Gibco-BRL and it is shown using two different UV-light intensities to allow its better visualization.

miRNA expression is generally examined to better understand their physiological function. In this study, the tissue expression profile was used to assess the mammary gland specificity of the new miRNAs. In addition these analyses provide additional evidence for the identification of a bona fide miRNA. Expression of the 33 new miRNAs cloned in this study was analysed using an adapted RT-PCR described by Shi and Chiang [42] and Ro *et al*. [43] and detailed in the Methods' section. Using this approach, we could detect expression for 22 of them in at least one of the 4 analysed tissues (lactating or involuting mouse mammary gland, brain, muscle and liver, Figure 3 and Table 3). Based upon the multi-tissues expression profiles, these new miRNAs have been grouped into 4 categories: undetected (11 miRNAs), ubiquitously expressed (10 miRNAs), mammary specific (4 miRNAs), and expression in several but not all tissues (8 miRNAs). Among the latter category, one is not expressed in the mammary gland stages used for the RT-PCR experiment (lactation and involution).

**Table 3:** Expression profile of the 33 new miRNA.

	Brain	Liver	Muscle	MG-L10d	MG-I03d
MG004	0	0	0	0	0
MG008	x	x	x	x	x
MG009	0	0	0	0	x
MG037	0	x	0	0	x
MG056	0	0	0	0	x
MG066	0	0	0	0	0
MG013	0	0	0	0	x
MG016	x	x	x	x	x
MG017	x	x	0	0	x
MG117	0	0	0	0	0
MG145	0	0	0	0	0
MG023	x	x	x	x	x
MG039	x	x	x	x	x
MG053	x	x	x	x	x
MG054	x	x	x	x	x
MG055	x	x	0	0	x
MG057	x	x	0	0	0
MG059	0	0	0	0	0
MG071	x	0	0	0	x
MG156	x	0	0	0	x
MG112	0	0	0	0	0
MG113	x	x	x	x	x
MG130	x	x	x	x	x
MG119	0	0	0	0	x
MG121	0	0	0	0	0
MG123	0	0	0	0	0
MG125	x	x	x	x	x
MG127	0	0	0	0	0
MG141	x	x	x	x	x
MG143	0	0	0	0	0
MG144	0	0	0	0	0
MG147	x	x	x	0	x
MG155	x	0	0	x	x

0: non detected, x: detected

The expression profile is the same for the 3 miRNAs present in the cluster MG113/MG130; MG141. The characterization of the undetected miRNAs was completed by studying their expression in some supplementary mammary gland stages (virgin 8 weeks, gestation day-18 or lactation day-4). Among the 11 miRNAs, only 2 have been detected (MG004 and MG123, data not shown). Among the 9 miRNAs undetected 3 correspond to variants of different families. We cannot exclude that these 3 variants are cloning artefact or that this lack of detection is due to individual variation for such miRNA families. In a same family all the variants are not expressed in the same tissues. A first analysis has allowed the identification of 4 mammary gland specific new miRNAs, to confirm this preliminary result their expression has been studied by RT-PCR on more tissues (heart, intestine, ovary, lung, spleen and kidney). Finally they were found not to be mammary gland specific: MG013 is expressed in all the tissues except kidney and heart; and MG056 is expressed in all the tissues except in ovary and kidney; MG119 have been detected in all tissues. MG009 was only found to be expressed in spleen and mammary gland.

In 11 cases the miRNA is detected in mammary gland in involution but not in lactation, a result in agreement with the expression profile of known miRNAs at different stages of the mammary gland biology obtained in our team (unpublished results). However, no correlation has been observed between the tissue-distribution of the miRNA expression (Table 3) and their evolutionary conservation (Table 2), or between their mammary expression profile (Table 3) and their conservation in mammals (Table 2). And the 6 miRNAs present only in the mouse genome are expressed in the 4 tissues tested. If in this study no mammary gland specific miRNA have been identified, several new miRNAs which are not ubiquitously expressed have been cloned.

### Functional validation of precursors detected by bioinformatics' analysis

**Figure 4:**
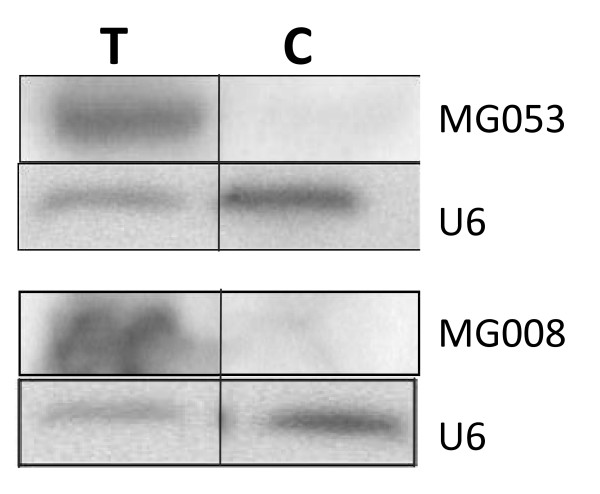
**Functional validation of precursors MG008-X and MG053-01**. Detection of miRNAs MG008 and MG053 after transfection of COS-7 cell with expression vectors vMG008 and vMG053 by Northern blot analysis. RNAs were extracted from cells transfected with expression vectors (T) and with the control empty vector (C), separated onto a 15% denaturing PAGE and transferred onto a nylon membrane Blots were hybridized with miRNA antisense oligonucleotides and a U6 probe used as an internal loading control.

The bioinformatics' analysis allowed the detection of potential precursors, but these results could not determine if these hairpin structures will be matured by RNase III, Drosha in the nucleus and Dicer in the cytoplasm. The use of expression vectors in cell culture, allowing the synthesis of hairpin structures that are matured into miRNAs, has already been demonstrated [44,45]. To test the functional validity of the precursors obtained by bioinformatics' analysis, we expressed them in transfected cells and checked for the presence of the mature miRNAs. The constructs carrying the precursors (MG008-X and MG053-01) were transiently transfected in COS-7 cells and the expression of the mature miRNAs (MG008 and MG053) was studied by Northern blot analysis (Figure 4). As a control the precursor of a known miRNA (let-7b) was used (data not shown). This approach could be used to validate precursor(s) of miRNA.

## Conclusion

In spite of the development of new tools, as the miRNA arrays, the cloning approaches remain the only strategy to identify miRNAs in a tissue at a specific stage. As the mammary gland is an evolutionary recent organ, it is a good candidate to search for new tissue-specific miRNAs. Our study provides evidence for the occurrence in this tissue of 3 already known miRNAs and of 33 new mouse miRNAs. Among the 33 new miRNAs, the expression of 24 of them was confirmed by RT-PCR analysis.

If none of them is mammary gland specific, some are not ubiquitous and are good candidates to further analyse their roles in the mammary gland biology.

One of the rules proposed by Ambros *et al*. [32] to characterize a miRNAs is the phylogenic conservation of the miRNA sequences. In our study, no correlation could be established between the expression and the evolutionary conservation of these new miRNAs. Our result is in agreement with the data obtained by Berezikov *et al*. [7] showing by comparing the miRNA content of human and chimpanzee brain that evolution of miRNAs is an ongoing process and that along with ancient, highly conserved miRNAs, there are a number of emerging miRNAs. Farh *et al*. [46] have suggested that the binding sites of miRNAs to 3'UTR do not necessarily have to be conserved among the different species. Therefore the miRNAs identified here that are conserved in non mammalian species could have also a specific role in the mammary gland, as the others. Expression in the different stages of the mammary gland biology and target identification of these new miRNAs will be critical for determining their functions.

This study has allowed the identification of 6 mouse specific miRNAs, reinforcing the yet unique ability of the cloning approach to identify such evolutionary not conserved miRNAs.

## Methods

### Collection of samples

The tissues were isolated from FVB/N mice. The mammary glands were collected at different stages: virgin 8 weeks, gestation 2-, 6- and 18-days, lactation 3- and 4-days and involution 1- and 3-day. For all pregnancy samples, day 0 of pregnancy is the day we observed the vaginal plug. Day 1 of involution was designated as 24 h after the removal of the pups. All mouse manipulations were done following the French Commission de Génie Génétique recommendations.

### Small RNA isolation and cloning

For the library construction, small RNAs were isolated using mirVana^TMP ^miRNA isolation kit (Ambion) according the manufacturer's instructions. Small RNA cloning was performed as described by Lagos-Quintana *et al*. [31] without the step of concatemerization of the PCR products. Briefly, 500 μg of RNA were size fractionated using 15% denaturing polyacrylamide gel electrophoresis (PAGE). Excised gel bands were homogenized in NaCl 0.3 M overnight at 4°C to solubilize RNA. 3' adapter (5' phosphorylated) was ligated to the RNA fraction in presence of T4 RNA ligase (Amersham-Pharmacia). The mix was size fractionated using 15% PAGE and a 5' adapter was ligated. The sample was again size fractionated using 15% PAGE before to be reverse transcribed using primer complementary to the 3' adapter sequence and PCR amplified using primers on both adapters. Amplified products were cloned using pGEM-T vector system I (Promega). Clones with inserts were sequenced.

### Bioinformatics' analysis

Small RNA sequences ranging between 18 and 25-nt in length obtained from the libraries were multi-aligned by CLUSTAL W software [47] to exclude redundant sequences [48]. The distinct sequences were used to search in miRBase [49] with BLASTN to identify conserved miRNA [50]. The sequences were mapped in the mouse genome from EnsEMBL mouse genome database [51]. A fragment of ~260 nucleotides genomic sequence flanking the small RNA at both 5' and 3' ends was used for predicting the secondary structure of the miRNA precursor (stem-loop formations) using the MFOLD program (version 3.2) [34,52]. If a sequence including a small RNA formed a stem-loop, if the small RNA size ranged from 18–25 nt and had not been registered in the miRBase, we classified it as a new miRNA. The sequences of the new miRNA candidates and their precursors were subjected to a BLASTN search against NCBI genomes to estimate the species conservation.

### miRNA expression analysis

Total RNAs were isolated from tissue samples and from COS cells using the RNA NOW kit (Biogentex inc.) according to the manufacturer's protocol with a small modification. The RNAs were precipitated at -20°C overnight by the addition of 3 volumes of ethanol. An equal-molar mix of total RNA from 3 different mice was used for reverse transcription.

Detection of miRNAs by RT-PCR has been realized after addition of linkers to the retro-transcribed RNA before amplification [42,43]. Briefly, 5 μg of total RNA were polyadenylated according to Ambion's protocol (polyA Polymerase, Ambion, Applied Biosystems, France). The polyadenylated RNAs were reverse transcribed with a polyT adapter composed of a polyT sequence and a universal oligonucleotide (5'CGAATTCTAGAGCTCGAGGCAGGCGACATGGCTGGCTAGTTAAGCTTGGTACCGAGCTCGGATCCACTAGTCC(T)_25_(ACG)(ACGT)3'). Detection was achieved by PCR using a set of primers composed of the universal primer corresponding to the 5' end of the polyT-adapter (5'CGAATTCTAGAGCTCGAGGCAGG3') and a primer specific of the miRNA sequence (Additional file 3). Reaction products were separated on 2% agarose gel. The detection of miRNA let-7c has been used as positive control and was indeed detected in all analysed samples (data not shown).

For Northern blot analysis, 20 μg of total RNA were fractionated using a 15% PAGE, transferred to Hybond-N+ membrane (Amersham) by capillarity. Blots were hybridized overnight at 55°C with radioactively [γ-^32^P]ATP labeled DNA oligonucleotide probe complementary to miRNA sequences in Phosphate buffer [53], washed twice with 2×SSC at 55°C, and exposed to Phosphor Screen and the StormScan software.

### Vector design

The precursor vectors contain the sequences design from MG008 and MG053 precursors (sequence in bold in Additional file 2). For MG053 precursor, the oligonucleotides sense (^5'^CGGGATCCCGAGCGCCGAATCCCCGCCGCGCGTCGCGGCGTG^3'^) and antisense (^5'^CGGGATCCCGGGTCTTCCGTACGCCACATTTCCCACGCCGCGACGCGCGGCGG^3'^) were annealed, filled with the Klenow fragment enzyme and digested by *Bam*HI. The resulting fragment was inserted into the *Bam*HI site of the pUHD10.3 plasmid [54]. The MG008 precursor fragment was obtained by PCR on mouse genomic DNA using the primer sense (^5'^CGGGATCCCGGTTTCAAAGTTTTGATAGGTTCTACGCATG^3'^) and antisense (^5'^CGGGATCCCGGCTTCAGCTTTGACTTTCAGAGCACTGGG^3'^) and digested by *Bam*HI before to be cloned into the *Bam*HI site of the pUHD10.3 plasmid. The orientation of the insert was characterized by PCR using the primer sense of the precursor and the primer of the plasmid design in each side of the cloning site (primer rtTA/1: ^5'^GATGCCCTGGAATTGACGAG^3' ^and primer glob/2: ^5'^TATAACATGAATTTTTCAATAGCG^3'^). In these vectors, the precursor is placed under the transcriptional regulation of the CMV promoter, already validated in COS-7 cell culture (personal communication).

### Cell culture experiments

COS-7 cell were cultured in Dulbecco's Modified Eagle's medium (DMEM) with addition of 10% fetal calf serum, 2 mM glutamine, penicillin 10 U/μl and streptomycin 100 U/μl at 37°C (5% of CO_2_). The expression vectors were transfected using the jetPEI™ cationic reagent (PolyPlus transfection) following the manufacturer's instructions. Cells were harvested 48 h after transfection.

## Authors' contributions

NS and LS were responsible for miRNA cloning and precursor validation in cell culture. JL analyzed the miRNA expression in different tissues by RT-PCR. GT has participated in miRNA cloning. SL has participated in expression vector constructions. JC has provided the mice used to collect tissue samples. JLV has participated in the project development and the manuscript elaboration. FLP was responsible for project development, sequence analysis and writes the paper. All contributing authors reviewed and approved the final copy of this manuscript.

## Supplementary Material

Additional file 1 **Distinct sequences identified by cloning from mouse mammary gland.** Click here for file

Additional file 2 **Predicted precursor structures of newly miRNAs**. The RNA secondary structure of the precursors was predicted using MFOLD program. The miRNA sequences are underlined; when variants have been detected the sequence extensions are in dotted lines. The chromosomal (Chr) locations of putative miRNA precursors are indicated. The bold nucleotides correspond to part of the precursors used to test their functionality. Click here for file

Additional file 3 **Primers specific of miRNAs used for expression characterisation of new miRNAs.** Click here for file

## References

[B1] Vasudevan S, Tong Y, Steitz JA (2007). Switching from repression to activation: microRNAs can up-regulate translation. Science.

[B2] Lim LP, Glasner ME, Yekta S, Burge CB, Bartel DP (2003). Vertebrate microRNA genes. Science.

[B3] Aravin A, Tuschl T (2005). Identification and characterization of small RNAs involved in RNA silencing. FEBS Lett.

[B4] Bentwich I (2005). Prediction and validation of microRNAs and their targets. FEBS Lett.

[B5] Pasquinelli AE, Reinhart BJ, Slack F, Martindale MQ, Kuroda MI, Maller B, Hayward DC, Ball EE, Degnan B, Muller P (2000). Conservation of the sequence and temporal expression of let-7 heterochronic regulatory RNA. Nature.

[B6] Bentwich I, Avniel A, Karov Y, Aharonov R, Gilad S, Barad O, Barzilai A, Einat P, Einav U, Meiri E (2005). Identification of hundreds of conserved and nonconserved human microRNAs. Nat Genet.

[B7] Berezikov E, Thuemmler F, van Laake LW, Kondova I, Bontrop R, Cuppen E, Plasterk RH (2006). Diversity of microRNAs in human and chimpanzee brain. Nat Genet.

[B8] Hutvagner G, McLachlan J, Pasquinelli AE, Balint E, Tuschl T, Zamore PD (2001). A cellular function for the RNA-interference enzyme Dicer in the maturation of the let-7 small temporal RNA. Science.

[B9] Ketting RF, Fischer SE, Bernstein E, Sijen T, Hannon GJ, Plasterk RH (2001). Dicer functions in RNA interference and in synthesis of small RNA involved in developmental timing in C. elegans. Genes Dev.

[B10] Bartel DP (2004). MicroRNAs: genomics, biogenesis, mechanism, and function. Cell.

[B11] Ambros V (2004). The functions of animal microRNAs. Nature.

[B12] Kim VN, Nam JW (2006). Genomics of microRNA. Trends Genet.

[B13] Landgraf P, Rusu M, Sheridan R, Sewer A, Iovino N, Aravin A, Pfeffer S, Rice A, Kamphorst AO, Landthaler M (2007). A mammalian microRNA expression atlas based on small RNA library sequencing. Cell.

[B14] Hennighausen L, Robinson GW (2005). Information networks in the mammary gland. Nat Rev Mol Cell Biol.

[B15] Watson CJ, Khaled WT (2008). Mammary development in the embryo and adult: a journey of morphogenesis and commitment. Development.

[B16] Wang V, Wu W (2007). MicroRNA: a new player in breast cancer development. Journal of Cancer Molecules.

[B17] Michael MZ, SM OC, van Holst Pellekaan NG, Young GP, James RJ (2003). Reduced accumulation of specific microRNAs in colorectal neoplasia. Mol Cancer Res.

[B18] Lu J, Getz G, Miska EA, Alvarez-Saavedra E, Lamb J, Peck D, Sweet-Cordero A, Ebert BL, Mak RH, Ferrando AA (2005). MicroRNA expression profiles classify human cancers. Nature.

[B19] Iorio MV, Ferracin M, Liu CG, Veronese A, Spizzo R, Sabbioni S, Magri E, Pedriali M, Fabbri M, Campiglio M (2005). MicroRNA gene expression deregulation in human breast cancer. Cancer Res.

[B20] Volinia S, Calin GA, Liu CG, Ambs S, Cimmino A, Petrocca F, Visone R, Iorio M, Roldo C, Ferracin M (2006). A microRNA expression signature of human solid tumors defines cancer gene targets. Proc Natl Acad Sci USA.

[B21] Si ML, Zhu S, Wu H, Lu Z, Wu F, Mo YY (2007). miR-21-mediated tumor growth. Oncogene.

[B22] Adams BD, Furneaux H, White BA (2007). The micro-ribonucleic acid (miRNA) miR-206 targets the human estrogen receptor-alpha (ERalpha) and represses ERalpha messenger RNA and protein expression in breast cancer cell lines. Mol Endocrinol.

[B23] Zhao JJ, Lin J, Yang H, Kong W, He L, Ma X, Coppola D, Cheng JQ (2008). MicroRNA-221/222 negatively regulates estrogen receptor alpha and is associated with tamoxifen resistance in breast cancer. J Biol Chem.

[B24] Mattie MD, Benz CC, Bowers J, Sensinger K, Wong L, Scott GK, Fedele V, Ginzinger D, Getts R, Haqq C (2006). Optimized high-throughput microRNA expression profiling provides novel biomarker assessment of clinical prostate and breast cancer biopsies. Mol Cancer.

[B25] Blenkiron C, Goldstein LD, Thorne NP, Spiteri I, Chin SF, Dunning MJ, Barbosa-Morais NL, Teschendorff AE, Green AR, Ellis IO (2007). MicroRNA expression profiling of human breast cancer identifies new markers of tumor subtype. Genome Biol.

[B26] Liu CG, Calin GA, Meloon B, Gamliel N, Sevignani C, Ferracin M, Dumitru CD, Shimizu M, Zupo S, Dono M (2004). An oligonucleotide microchip for genome-wide microRNA profiling in human and mouse tissues. Proc Natl Acad Sci USA.

[B27] Wang C, Li Q (2007). Identification of differentially expressed microRNAs during the development of Chinese murine mammary gland. J Genet Genomics.

[B28] Silveri L, Tilly G, Vilotte JL, Le Provost F (2006). MicroRNA involvement in mammary gland development and breast cancer. Reprod Nutr Dev.

[B29] Gu Z, Eleswarapu S, Jiang H (2007). Identification and characterization of microRNAs from the bovine adipose tissue and mammary gland. FEBS Lett.

[B30] Ibarra I, Erlich Y, Muthuswamy SK, Sachidanandam R, Hannon GJ (2007). A role for microRNAs in maintenance of mouse mammary epithelial progenitor cells. Genes Dev.

[B31] Lagos-Quintana M, Rauhut R, Lendeckel W, Tuschl T (2001). Identification of novel genes coding for small expressed RNAs. Science.

[B32] Ambros V, Bartel B, Bartel DP, Burge CB, Carrington JC, Chen X, Dreyfuss G, Eddy SR, Griffiths-Jones S, Marshall M (2003). A uniform system for microRNA annotation. Rna.

[B33] Blow MJ, Grocock RJ, van Dongen S, Enright AJ, Dicks E, Futreal PA, Wooster R, Stratton MR (2006). RNA editing of human microRNAs. Genome Biol.

[B34] Zuker M (2003). Mfold web server for nucleic acid folding and hybridization prediction. Nucleic Acids Res.

[B35] Kim J, Cho IS, Hong JS, Choi YK, Kim H, Lee YS (2008). Identification and characterization of new microRNAs from pig. Mamm Genome.

[B36] Ruby JG, Jan C, Player C, Axtell MJ, Lee W, Nusbaum C, Ge H, Bartel DP (2006). Large-scale sequencing reveals 21U-RNAs and additional microRNAs and endogenous siRNAs in C. elegans. Cell.

[B37] Ruby JG, Stark A, Johnston WK, Kellis M, Bartel DP, Lai EC (2007). Evolution, biogenesis, expression, and target predictions of a substantially expanded set of Drosophila microRNAs. Genome Res.

[B38] Ro S, Song R, Park C, Zheng H, Sanders KM, Yan W (2007). Cloning and expression profiling of small RNAs expressed in the mouse ovary. Rna.

[B39] Mishima T, Takizawa T, Luo SS, Ishibashi O, Kawahigashi Y, Mizuguchi Y, Ishikawa T, Mori M, Kanda T, Goto T, Takizawa T (2008). MicroRNA cloning analysis reveals sex differences in microRNA expression profiles between adult mouse testis and ovary. Reproduction.

[B40] Ro S, Park C, Sanders KM, McCarrey JR, Yan W (2007). Cloning and expression profiling of testis-expressed microRNAs. Dev Biol.

[B41] Altuvia Y, Landgraf P, Lithwick G, Elefant N, Pfeffer S, Aravin A, Brownstein MJ, Tuschl T, Margalit H (2005). Clustering and conservation patterns of human microRNAs. Nucleic Acids Res.

[B42] Shi R, Chiang VL (2005). Facile means for quantifying microRNA expression by real-time PCR. Biotechniques.

[B43] Ro S, Park C, Jin J, Sanders KM, Yan W (2006). A PCR-based method for detection and quantification of small RNAs. Biochem Biophys Res Commun.

[B44] Zeng Y, Wagner EJ, Cullen BR (2002). Both natural and designed micro RNAs can inhibit the expression of cognate mRNAs when expressed in human cells. Mol Cell.

[B45] Silva JM, Li MZ, Chang K, Ge W, Golding MC, Rickles RJ, Siolas D, Hu G, Paddison PJ, Schlabach MR (2005). Second-generation shRNA libraries covering the mouse and human genomes. Nat Genet.

[B46] Farh KK, Grimson A, Jan C, Lewis BP, Johnston WK, Lim LP, Burge CB, Bartel DP (2005). The widespread impact of mammalian MicroRNAs on mRNA repression and evolution. Science.

[B47] EMBL-EBI. http://www.ebi.ac.uk/clustalw/.

[B48] Thompson JD, Higgins DG, Gibson TJ (1994). CLUSTAL W: improving the sensitivity of progressive multiple sequence alignment through sequence weighting, position-specific gap penalties and weight matrix choice. Nucleic Acids Res.

[B49] miRBase::Sequences. http://microrna.sanger.ac.uk/.

[B50] Griffiths-Jones S, Saini HK, van Dongen S, Enright AJ (2008). miRBase: tools for microRNA genomics. Nucleic Acids Res.

[B51] Ensembl. http://www.ensembl.org/Mus_musculus/.

[B52] RNAfold web server. http://rna.tbi.univie.ac.at/cgi-bin/RNAfold.cgi.

[B53] Church GM, Gilbert W (1984). Genomic sequencing. Proc Natl Acad Sci USA.

[B54] Gossen M, Bujard H (1992). Tight control of gene expression in mammalian cells by tetracycline-responsive promoters. Proc Natl Acad Sci USA.

