# Supplementary material for: Identification and characterization of new miRNAs cloned from normal mouse mammary gland
Source: BMC Genomics. 2009 Apr 7;10:149. doi: 10.1186/1471-2164-10-149 (PMC2683868; doi:10.1186/1471-2164-10-149)
Supplement: Additional file 2 — Predicted precursor structures of newly miRNAs. The RNA secondary structure of the precursors was predicted using MFOLD program. The miRNA sequences are underlined; when variants have been detected the sequence extensions are in dotted lines. The chromosomal (Chr) locations of putative miRNA precursors are indicated. The bold nucleotides correspond to part of the precursors used to test their functionality. [file 1471-2164-10-149-S2.doc]

**MG004/MG008-X** Chr X: 113400923-113401101, intergenic, 179 nt

**AAA**  **AGG**  **UAC GCUAC A- UG UAACA UU- AA A ---- AUACAACGAAAU**

--**GUUUC** **GUUUUGAU UUC GCAU UAGAAA G UU GUCA CC GCUGUG GUC UGUGUG A**

UAAAG **CGAAACUG AAG CGUG GUCUUU U AG CAGU GG CGGCGC CGG AUACGC C**

AAG **A-- UCU ACCC- AG GU CG--- UGU G- C UGUC AACAUAGAAUUU**

**MG009/MG037/MG056/MG066-06** Chr 6: 3151217-3151307, intergenic, 91 nt

- C- ---- CGAA GUU--- AA

UUAGCUG AGUGUCC GCGG GGCC GC UACUUUGA \

GAUCGAC UCAUAGG CGCC CCGG CG GUGAGAUU A

G UC GAGC A--- AAACUU AA

**MG009/MG037/MG056/MG066-17** Chr 17: 39984073-39984163, intergenic, 95 nt

A- C- ---- CGAA GUU--- AA

UCUUAGCUG GUGUCC GCGG GGCC GC UACUUUGA \

AGGAUCGAC CAUAGG CGCC CCGG CG GUGAGAUU A

GC UC GAGC A--- AAACUU AA

**MG013-11** Chr 11: 115275130-115275223, intergenic, 94 nt

AUCA------- G ------- - UUCGUG G U

GAAGAUUGAGG UUC GAG UCCC GUC AAAUG U

CUUUUAAUUCU AAG CUC AGGG UAG UUUAC U

GUUACGAUUAA - UAACUUU U ------ G U

**MG016-01** Chr 1: 169270471-169270582, intergenic, 55 nt

GUU CU AAAC U GGA

GU UGGG GGGG GCGGCC A

CA GCCC CUCC CGCCGG A

--- CU GCU- - GGG

**MG016-04** Chr 4:138894195-138894304, intergenic, 57 nt

-GUU C- - AAC U GGA

CGU UUGG GA GGGG GCGGCC A

GCA GACC CU CUCC CGCCGG A

--- CU G --- - GGG

**MG016-09a** Chr 9: 56071503-56071642, intronic, 90 nt

GAUCC GCGG A UU - - AAA GU - GGA

GGU AG GCCA GGU CU UGGG CGGG G CGGCC A

CCA UC CGGU CCA GG GCCC GCUC C GCCGG A

A---- AA-- G -- A U --- UG C GGG

**MG016-13 and MG016-16** Chr 13: 9833686-9833797, intergenic ;

Chr 16: 11144192-11144303, intronic, 62 nt

GUU CU AAAC U GGA

CGU UGGG GGGG GCGGCC A

GCA GCCC CUCC CGCCGG A

--- CU GCU- - GGG

**MG017/MG117/MG145-06b** Chr 6: 145881295-145881404, intronic, 105 nt

CCUCC A- -- AG - CUC CUU C ACGUACGC

GA GUUUC CCUCAGGAU CUGG CG UCGCU UU CCG \

CU CAAAG GGGGUUCUG GAUU GU AGCGA AA GGC A

CAACU AG CC GA A A-- --- U CUAUUUUG

**MG017/MG117/MG145-14** Chr 14: 105115137-105115246, intergenic, 100 nt

CUCCGAA -- AG - UC CUU C ACGUACGC

GUUUC CCUCAGGAU CUGG CGU UCGCU UU CCG \

CAAAG GGGGUUCUG GAUU GUA AGCGA AA GGC A

ACUCUAG CC GA A -- --- U CUAUUUUG

**MG017/MG117/MG145-17** Chr 17, NW_001030622.1: 16678358-16678257, intergenic, 95 nt

----GAA -- AG - CUC AU C ACGCAUGC

GUUUC CCUCAGGAU CUGG CG UCG CUUU CCG \

CAAAG GGGGUUCUG GAUU GU AGC GAAA GGC A

ACUCUAG CC GA A A-- -- U CUAUUUUG

**MG023-09** Chr 9: 104328443-104328517, intergenic, 75 nt

- - GAGU G -- UAA

CCGC GUAUC CAGUCAG CA UUAGGA AGUUU \

GGUG UGUAG GUCAGUU GU GGUCUU UUAAG G

G A ---- G AA UCU

**MG039-X** Chr X: 70237418-70237483, 5’UTR, 66 nt

AUAC -- CU - C

GUAGU CUC CUUCCUG GC CUUGUUGG U

CGUUA GAG GAGGGAC CG GGGCGGCC U

C--- AU CC A G

**MG053-01** Chr 1, AAHY01156705: 475-590, intergenic, 116 nt

--- - cg c----- u **uccgua acauu a a**

ccc ca agcggcg cggggag g**ggucu** **cgcc uccc cgccgcg** **\**

ggg gu uugccgc gucccuu cucggg **gcgg aggg gcggcgc c**

uaa a ca ucacuu - **uc---- cuu-- - g**

**MG054-03b** Chr 3: 120952687-120952772, intronic, 86 nt

AA CCAA AC U- - AA AAC

CCACAG GGGA GGGCU GG CGG UCAGCGGGG G

GGUGUC CUCU UCUGA UC GUU AGUUGUCCC A

CC ---- GA UC A CG AGA

**MG055-01a, MG055-01b and MG055-01c**

Chr 1: 172996751-172996851, 173004422-173004522,173011996-173012096,intergenic, 70 nt

C----- A A A- G UU AGU UA-- UC G A

AGG GAC GGGC UG GUGG C GG GAAUUC GCCU CC C

UCC CUG CCUG AC UACC G CC CUUGGG CGGA GG G

UGGUUU - A AG G CG C-- UUAG CC G C

**MG055-06** Chr 6: 148357102-148357169, intronic, 68 nt

------- - U G- AAUU

GAGGGCAUGGG UG GU CA UGGUAG \

CUCUUGUGCUC AC CG GU ACCGUC C

UUUAGUU U U GA CGCU

**MG057-04** Chr 4: 139523441-139523535, exonic, 95 nt

AG GA AGG CU AUUUGGACAU C-- AG

AAG GCAG GCCAGGA GGC GCAGCUGC CAGCA G

UUC CGUC UGGUCCU UCG CGUCGACG GUCGU U

-- GG AA- CG ---------- CCU GG

**MG059-16** Chr 16: 39573327-39573386, intergenic, 60 nt

AU------- - UGACC - GCU

AUUCUU GAACCCC UC CU \

UAAGAA CUUGGGG AG GA U

UAAUACGUC U U---- U ACU

**MG071/MG156-02** Chr 2: 118872509-118872687, intergenic, 179 nt

U AA CGCACU U- ACAAAA G CCAU - UG A C- CUGU A C AUAG

GUGU UGCAGAGU ACAAU GUUGGC UCCGU UGGC ACUU GGU AU CGUC CAG GCU GUCU CU CCU \

UACG ACGUCUCG UGUUA UAAUCG AGGUA AUUG UGAG CCG UG GCAG GUC CGA CAGA GA GGA C

C -- CC---- UC GCGGA- G ---- G GA G UU U--- - A CCAU

**MG071-13a** Chr 13: 22086051-22086146, intergenic, 96 nt

A--- G - --------- A - UA - CUG

GAGA GACGA GGUGG CCG GUG GU AG GCGAUGGA \

CUCU CUGCU CCACC GGU CGC CG UC UGUUACCU C

AUAC G U CUAAGCUUG G A UC G AAU

**MG071/MG156-19** Chr 19: 5038299-5038402, 3’UTR, 104 nt

AU UGU -- GUGG A - U - CUG

AGGAUC CG GUGGGA CGAG CCG GUG GU AAG GCGAUGGA \

UCCUGG GC UACCCU GCUU GGU CGC CG UUC UGUUACCU C

CU UCC AA G--- G A U G AAU

**MG112-07** Chr 7: 71138847-71138901, intergenic, 55 nt

A A- GG AA---- AGU

GCU GCCG UUGAG CUCC U

CGA UGGU AACUC GGGG G

- GA A- GAUUAG AG

**MG113/MG130-12** Chr 12: 100883257-100883333, intronic, 77 nt

AGAUAAUU UA ACA CAAG AAC AUG

G GUU CGGAC GAGUCU GC C

C CGA GCCUG CUCGGG UG A

CGCGGCGC GC AA- ---- GAC AGC

**MG119-18** Chr 18: 85867321-85867373, intergenic, 53 nt

UCCACAGG -- GUGG GC

UGCAGA UCUUG UAGUA \

ACGUUU AGAGC AUUAU A

-------- CA AA-- AA

**MG121-13b** Chr 13: 44844997-44845085, intronic, 89 nt

AAGGUA U A---- CACA AUAA A

GGAGG GUCAGAAA GUUGC GGG UUGGCUA U

UUUCC UAGUUUUU CAACG CCC AACCGGU G

AG---- - CGAUG AUA- GUG- G

**MG123-15** Chr 15: 4463688-4463791, intronic, 104 nt

AA UUU C UG G AGC- AA

AUAUAA AUACA AGGUG AGCGUUGG GUAUAGU GUG AUAAA U

UAUAUU UAUGU UUUAU UUGUAACC UAUAUUA CAU UAUUU A

C- CU- - -- A AAAU AC

**MG123-Xa** Chr X: 38579050-38579135, intergenic, 86 nt

AC --- -- AGU----- G AUA GCC

AACC UGCGUUGGU GGUAU GGU AGC GCU U

UUGG ACGCAACCG CCAUA CCA UUG CGA U

-- AGU AC GUUUGGAC G A-- ACC

**MG125-16** Chr 16: 33002031-33002114, intronic, 84 nt

CG AAGCAAGGU - UAGUA UG A G

UCU GAAGCU CAG GCCUGGU CU G UGG A

AGG UUUCGG GUC UGGGCCA GG C GCC G

UU AU------- G UAA-- GU C A

**MG127-01** Chr 1: 115800566-115800710, intergenic, 145 nt

A GUCC CU A GCGAAACCACA AGGGAA U- G A- GAAA

UUCCCACU CUAC ACU UUCA GCCA CGGGCU GGU GA UCAGCGGGG \

GAGGGUGA GAUG UGG GAGU UGGU GUCUGA UCA UU AGUUGUCCC G

- AUAA -- A ACAGAGAAG-- AUG--- UC G CG AGAA

**MG141-03** Chr 3: 37380281-37380376,intronic, 76 nt

GAAGAG C - CC GU AUC AG--- - AG C

CCCGGC CGG GG CGAG GGG CCG GC CUCUCC UC G

GGGCCG GCC CC GCUC CCC GGC CG GGGAGG AG C

------ C G C- UG --- CACCA C G- C

**MG141-12** Chr 12: 100883323-100883403, intronic, 81 nt

G- CAAA- - GAA C- - G

CGCC UGGCG GA AGGU GGG CCCGCC CGG G

GCGG GCCGC CU UCCG CCC GGGUGG GCC G

GA CUGAC C GAG UA A C

**MG141-15** Chr 15: 86025202-86025279, intergenic, 82 nt

G-- CAA A-- GAA C- - G

GCGC UGGCG UG AGGU GGG CCCGCC CGG G

CGCG GCCGU AC UCCG CCC GGGUGG GCC G

GGA CUG CUC GAG UA A C

**MG143-08** Chr 8: 15520059-15520129, intergenic, 66 nt

GUGGAAUG A G--- A UG - C GU

CG GU CCU G G GCCA UUUUG \

GC CA GGG C C CGGU AAGAC A

AGUAUCAA - AGUA - GU G C GA

**MG144-04d** Chr 4: 58950891-58950984, intergenic, 94 nt

AA A CAU- C- ACA AUCCCA A

UCAUGUCUAU GU ACCA CCUGA UGCUUG UCUG U

GGUGCAGAUA CG UGGU GGGCU ACGAGC AGAC C

-- - UAAU CC GGG CGA--- U

**MG144-14** Chr 14: 78710956-78711107, intergenic, 152 nt

AUCU CUAAGCA- CU- U CA- UGGG UUG --- A GCAA

CGGAAG GGGUCGGGC GGU AG CUUGGA AGAAACG AGGCA GGCUAG GU U

GCCUUC CUCAGUCCG UCA UC GAACCU UCUUUGU UUCGU CCGGUC CA U

G--- UUCCUUUA UGU C CUC UGAG GA- UAC A ACCC

**MG147-09** Chr 9: 3258577-3258630, miRNA non-fully characterized, 54 nt

AAG A AG GU -- G- A

UGG GA G U CCAUGU AAC A

ACC CU C A GGUACA UUG C

A-- - GA UG CU AG A

**MG155-17** Chr 17: 26011994-26012053, intergenic, 59 nt

ACAAUUCGU - GACUG CU C

AUGCC CUACG CAGU CGCU A

UAUGG GAUGU GUCG GCGA A

--------- U G---- CC C
